# Influence of Coriander Seed Powder on Texture, Rheological Properties, and Sensory Quality of Spoonable Yoghurt

**DOI:** 10.3390/foods14244315

**Published:** 2025-12-15

**Authors:** Wan-Ying Zhang, Yang Sun, Hai-Bo Lu, Yue-Yuan Lu, Guo-Jun Du, Chun-Li Song, Jian Ren, Li-Ying Bo, Jing-Jing An, Meng Wang

**Affiliations:** 1 Faculty of Food Quality and Safety, Qiqihar University, Qiqihar 161006, China; 18745150721@163.com (W.-Y.Z.); xinyang_1021@163.com (Y.S.); 15604891260@163.com (H.-B.L.); 18954269506@163.com (Y.-Y.L.); dgj2222@163.com (G.-J.D.); songchunlilily@sina.com (C.-L.S.); renjian1970789@163.com (J.R.); 2 Tianjin Institute of Industrial Biotechnology, Chinese Academy of Sciences, Tianjin 300308, China; wangmeng@tib.cas.cn; 3 Haihe Laboratory of Synthetic Biology, Tianjin 300308, China

**Keywords:** coriander seed powder, solidified yogurt, texture, rheological properties, in vitro protein

## Abstract

This study investigated the effects of various concentrations of coriander seed powder (CSP) (0.1%, 0.3%, and 0.5%) on the fermentation performance, whey precipitation rate, texture, rheological properties, microstructure, and sensory characteristics of spoonable yoghurt stored at 4 °C for 21 days. The aim was to evaluate the impact of CSP on spoonable yoghurt quality and its health-promoting properties. The results revealed that the addition of CSP led to a significant decrease in pH and an increase in titratable acidity. Furthermore, the whey precipitation rate in the CSP-enriched yogurt sample was clearly lower than that of the control group (*p* < 0.05). The syneresis of the yogurt sample with 0.3% CSP decreased by 21.14 on the 21st day, compared to the control group (49.64%) (*p* < 0.05). This was accompanied by a reduction in apparent viscosity and an increase in the viscoelastic modulus. Meanwhile, the texture of the yogurt sample remains more stable, with the best sensory acceptance in the yogurt sample containing 0.3% CSP. As the concentration of coriander seed powder level increased, antioxidant activity, digestibility, and small molecule phenolic level of the yogurt samples obviously improved (*p* < 0.05). Yogurt with 0.3% CSP still showed significantly enhanced antioxidant capacity during the 21-day storage period. The DPPH-radical scavenging rate increased by 5.22% compared to the control group (*p* < 0.05). Similarly, the ABTS+ clearance activity increased by 12.52% (47.06% compared to 34.54% in the control group, *p* < 0.05). In the 0.3% CSP yogurt group, the total phenolic content reached 5.33 mg GAE/100 g, an increase of 1.85 compared to the control sample (3.48 mg GAE/100 g) (*p* < 0.05). The in vitro protein digestibility of the yogurt samples containing 0.3% CSP clearly increased by 12.65% (*p* < 0.05). In summary, the yogurt sample supplemented with 0.3% CSP demonstrated optimal sensory quality characteristics. Coriander seed powder may be used as a beneficial ingredient containing rich active substances to enhance the quality of spoonable yoghurt products.

## 1. Introduction

With the progressively consumer requirement for functionality food, nutritious foods have obtained significant attention [1]. The term nutrition food (NF) originated in Japan in the 1980s, aimed at addressing the challenges of an aging population and increasing government healthcare costs [2]. Nutritious foods contain bioactive ingredients, such as fiber, probiotics, vitamins, and antioxidant substances, which are associated with disease prevention and the improvement of overall health [2,3]. Yogurt is a highly popular and widely consumed fermentation milk product, considered a functional food that serves consumers with various health benefits [4]. It is generally supposed to be rich in vitamins, calcium, protein, and probiotics, with a series of health benefits, comprising enhanced nutritional value (B vitamins, magnesium, calcium, zinc, and phosphorus), improved lactose digestion, and strengthened immunity [5,6]. Correspondingly, adding natural bioactive components to yogurt products has become an effective strategy to improve their nutrition and overall quality [7]. Natural substances, e.g., fruits, vegetables, herbs, and spices, are abundant in bioactive compounds like anthocyanins, vitamins, flavonoids, and minerals. These compounds exhibit antioxidant, regulatory immunity, anti-inflammatory, and other bioactive properties that can further fortify the nutritional value of yogurt [8,9]. However, conventional yogurt products often lack such bioactive compounds (e.g., phenolics, flavonoids, anthocyanins, and iron) [10,11]. Thus, fermented dairy products supplemented with natural ingredients can confer additional health benefits, including anti-diabetic, anti-cancer, antibacterial, and anti-obesity functions.

In recent years, numerous researchers have dedicated their efforts to exploring future food rich in polyphenols, abundant in excellent antioxidants, and featuring anti-inflammatory activities. For instance, adding apple pomace to yogurt is a good example. Apples are rich in polyphenols and flavonoids, which have been proven to enhance the stability of yogurt products. Particularly at higher concentrations, it can improve the gel hardness and elasticity of yogurt, with obviously strengthened textural characteristics [12,13]. Similarly, date press cake can obviously enhance yogurt functionality. Its addition clearly reduces syneresis and increases viscosity, thus heightening overall sensory quality and stability of yogurt [14]. Another type of yogurt treatment is to add carrot juice, which contributes to a firm and stable texture in yogurt products [15]. Another study also confirmed that the addition of rosehip seed powder increased the total phenol content of yogurt. The total phenol content in yogurt with 3% fruit residue rose from 12.80 ± 0.14% to 15.45 ± 0.01%, compared with the control yogurt [16]. These polyphenol-rich substances help neutralize free radicals, reduce oxidative stress, and thereby lower the risk of chronic diseases [17]. Saatloo et al.’s research explored the effects of different amounts of added coriander seed powder on the activity, physicochemical properties, and survival rate of *Bifidobacterium* and *Lactobacillus* during yogurt storage. However, this study did not discuss the effects of coriander seed powder on the total phenol content, antioxidant activity, in vitro digestibility, and microstructure of yogurt [18]. Coriander seeds are not only used as spices and seasonings, but are also of major value in sectors such as medicine, cosmetics, and the food industry [19]. For example, coriander seed extract can be used for developing functional products, dietary supplements, and cosmetic products. Overall, coriander seeds are a versatile natural plant with numerous health benefits [20]. Through scientific research and rational utilization, coriander seeds are expected to play an increasingly key role in the food and healthcare industries [17]. The goal of the research is to develop a yogurt product with improved nutrition value and storage quality, while also satisfying consumer demands from a sensory perspective. To attain this goal, the influences of adding coriander seed powder (CSP) at different levels of 0.1%, 0.3%, and 0.5% on the microstructure, rheology properties, texture, and sensory qualities of the yogurt samples were investigated in order to identify the optimal addition concentration of CSP.

## 2. Materials and Methods

### 2.1. Materials and Reagents

Whole milk powder was obtained from Inner Mongolia Yili Group Co., Ltd. (Hohhot, China) The yogurt starter, sourced from Beijing (Beijing Chuanxiu Technology Co., Ltd., Beijing, China), contained *Streptococcus thermophilus* and *Lactobacillus delbrueckii* subsp. *bulgariacus*. Coriander seeds were procured from the local market. All other chemical reagents used in this experiment were analytical grade.

### 2.2. Preparation of CSP and Milk Mixture

Coriander seeds were ground with an ultra-fine grinder for 15 min. The obtained powder was sifted through a 100-mesh sieve (<75 μm) and stored in airtight containers until experimental use. A base solution was prepared by dissolving 12% (*w*/*w*) whole milk powder and 8% (*w*/*w*) sucrose in purified water at 65 °C. Coriander seed powder was subsequently added to the base solution at different concentrations of 0.1%, 0.3%, and 0.5% (*w*/*w*), respectively. The mixture was homogenized at 100 MPa with a homogenizer (Panda Plus 2000, GEA Niro Soavi S.p.A., Parma, Italy). After the homogenization treatment, the milk was heated to 90 °C for 10 min for pasteurization, then cooled to 42 °C and covered with sterile sealing film. Finally, the milk was inoculated with a fermentation medium culture containing 0.2% (*w*/*v*) concentration of *Streptococcus thermophilus* and *Lactobacillus delbrueckii. bulgariacus*. The control sample without coriander seed powder also underwent the same processing.

### 2.3. Yogurt Fermentation

The set yogurt was prepared according to the method described by Megrous et al. [21], with minor modifications. Briefly, the inoculated milk mixture was distributed into 50 mL beakers, sealed, and fermented at 42 °C for 5 h. After fermentation, all samples were immediately transferred to a refrigerator and stored at 4 °C for 24 h before further analysis.

### 2.4. Detection of pH and Titratable Acidity of the Yogurt Samples

The pH of each yogurt sample was measured every 1 h using a digital pH meter (Saidoris Scientific Instruments, Beijing, China) during fermentation processing on the 1st, 7th, 14th, and 21st. After calibration, the pH electrode was used for determination by the direct immersion method. The acidity is determined by the titration method with minor modifications [22]. Briefly, 5 g of yogurt was dissolved in 20 mL of purified water at room temperature, and 0.5% (*w*/*v*) phenolphthalein served as an indicator. All samples were then titrated with NaOH solution (0.1 N) to test the titratable acidity (TA). The endpoint was reached when a continuous light pink color persisted. The TA value was expressed as the volume of NaOH consumed by 100 g of the yogurt sample (mL). All determinations were performed in triplicate.

### 2.5. Measurement of Syneresis of All Samples

The determination of the syneresis change in the yogurt samples was conducted based on the method provided by Parvarei et al. [22,23]. Briefly, 5 mL yogurt samples were placed into 10 mL centrifuge tubes for constant temperature fermentation and storage. The yogurt samples were stored for 1, 7, 14, and 21 days, then removed for testing. The total mass of each centrifuge tube and yogurt sample was recorded prior to centrifugation. Then, the centrifuge tubes containing the yogurt were centrifuged for 10 min at 9050× *g*. The supernatant was carefully collected and weighed. Syneresis was computed as the percentage of the supernatant weight relative to the initial weight of the yogurt sample.

### 2.6. Texture Assessment of the Yogurt Samples

Texture analysis was performed according to Jooyandeh’s method [24], with minor modifications. Spoonable yoghurt samples were tested at room temperature with a texture analyzer instrument (QTS-25; AMETEK Brookfield, Middleboro, MA, USA) with a 35 mm diameter cylindrical probe. Test parameters included probe speed (1 mm/s), trigger force (0.15 N), and penetration distance (30 mm), achieving 50% compression. Texture analysis was conducted using a double penetration method. Texture data analysis was implemented using Texture Lab Pro software (version is 1.18-408 15/10/13, Beijing Yingsheng Hengtai Technology Co., Ltd., Beijing, China, Country of Origin: Spain).

### 2.7. Rheological Determination of the Yogurt

The rheology properties of all samples were comprehensively characterized at room temperature, following Ge et al.’s method [25], with a focus on key parameters including viscoelasticity and viscosity. Measurement was performed with a Kinexus Pro^+^ rotational rheometer (Malvern Instruments, Worcestershire, UK). The measurement was performed using a cone-plate geometry (cone angle: 4°, gap: 0.15 mm, diameter: 40 mm) at room temperature. Approximately 2 g of the sample was evenly spread onto the lower plate, with excess material trimmed from the edges using a dedicated scraper. Apparent viscosity was measured under 0.1 s^−1^ to 100 s^−1^ shear rate. In order to further investigate the viscoelastic performance, frequency sweep assays were conducted at a fixed strain (0.5%) under the angular frequency condition (0.1–10 Hz). During these tests, the system automatically recorded the storage modulus (G′) and loss modulus (G′′) to assess the structural elasticity and flow behavior of the yogurt.

### 2.8. Determination of the Antioxidant Activity of the Yogurt Samples

The DPPH-radical scavenging capacity was tested using a modified method from Zhu et al. [26]. Briefly, 3 mL of the yogurt sample was mixed with DPPH (3 mL, 0.1 mmol/L in ethanol) and vortexed. The mixtures were incubated in the dark at room temperature for 30 min, centrifuged at 4000× *g* for 10 min. The absorbance value of the supernatant was tested at 517 nm. The clearance rate was calculated according to the formula:

$$\mathrm{DPPH}\;\mathrm{Clearance}\;\mathrm{Rate}\;\%\;=\;[1\;-\;\frac{A_{1}\;-\;A_{2}}{A_{0}}]\;\times \;100\%$$

where A_0_ represents the absorbance value of the control, A_1_ stands for the absorbance value of the sample, and A_2_ shows the absorbance of the sample-ethanol mixture.

The determination method of ABTS-free-radical scavenging activity was detected based on the modified method provided by Stella et al. [27]. Briefly, 40 μg/mL of the ABTS-radical stock solution was diluted with PBS and used as the ABTS-radical working solution. A sample solution with a concentration of 1 mg/mL was prepared. Then, 100 μL of the sample solution was added to a 96-well plate, followed by 100 μL of ABTS-free-radical working solution. The resulting absorbance is denoted as A_1_. Phosphate-buffered saline (PBS) was used instead of the sample as the control, and the absorbance was recorded as A_0_. After stirring, the sample was allowed to stand for 10 min, and the absorbance was measured at 734 nm.

$$\mathrm{ABTS}\cdot \mathrm{clearance}\;\mathrm{rate}\;\%=[1\;-\frac{A_{0}\;{\;-\;A}_{1}}{A_{0}}]\;\times \;100\%$$

### 2.9. Determination of Content of Bioactive Substances (Phenolic and Flavonoids Content) of the Yogurt

The coriander seed powder yogurt extract was manufactured following the method obtained by Anwar et al. [28]. Briefly, a 10 g yogurt sample with coriander seed powder was homogenized using 15 mL of acidified methanol with 0.05 mL concentrated HCl in a centrifuge tube. These mixtures were centrifuged at 5000× *g* at 4 °C for 20 min, and then the supernatant liquid was saved for subsequent assessment.

The total phenolic content of the yogurt sample with the coriander seed powder was tested with the Folin–Ciocalteu method, as published by Anwar et al. [28]. Briefly, 0.3 mL of the different concentrations of coriander seed powder yogurt extract was mixed with Folin–Ciocalteu analoids (0.3 mL) and incubated for 5 min at room temperature. Then, 75 g/L sodium carbonate solution (3 mL) was added. After 5 min of incubation treatment, the mixture was diluted to 10 mL with purified water and further incubated at 50 °C in the dark for 5 min. Absorbance value was determined at 750 nm. The total phenolic content of all samples was computed based on a gallic acid standard curve and is shown as mg gallic acid equals per 100 g of sample (mg GAE/100 g).

The colorimetric method was used to assess the total flavonoid content of coriander seed powder yogurt [28]. In brief, the coriander seed powder yogurt extract at various concentrations was diluted six-fold with distilled water. Then, 3 mL of the diluted extract was mixed with 0.15 mL of a 5% (*w*/*v*) sodium nitrite solution. After incubation treatment for 5 min, 0.3 mL aluminum chloride agentia was added. After another incubation treatment (5 min), 1 mL of sodium hydroxide solution (1 M, NaOH) was added. The mixture was then diluted to 5 mL with distilled water, vortexed completely, and the absorbance was tested at 510 nm. Quantification was conducted according to the rutin standard curve, and the results were shown as rutin equivalents (mg RE/100 g sample).

### 2.10. Determination of Protein Digestibility In Vitro of All Samples

The protein digestibility test was conducted on the basis of Oliveira’s method with a few modifications [29]. Equal samples (10 g) were shifted to individual centrifuge tubes (50 mL), diluted with NaCl reagent (0.9%, 10 mL), and the pH was adjusted to 2.0 to perform the gastric simulation assessment. During the simulation of the gastric digestion process, the pH was constantly maintained at 2.0 using 1 M HCl, and the simulated gastric solution was added (0.05 mL per mL digest). Then the mixture was incubated at 37 °C and shaken at 100 rpm for 60 min. The undigested samples and final gastrointestinal digesta were centrifuged at a speed of 8000× *g* (4 °C, 20 min), and then the protein level was quantified using the Bradford assay [30]. After centrifugation, the supernatant was diluted to 100 mL. Then, 1 mL of the diluted solution was mixed with 5 mL of Coomassie Brilliant Blue G-250 reagent. After incubation, the absorbance was measured at 595 nm. Protein digestibility was calculated using the formula:

$$\mathrm{Digestibility}\;\left(\%\right)=\frac{\begin{array}{c} Soluble\;protein\;in\;digesta \end{array}}{\mathrm{Total}\;\mathrm{protein}\;\mathrm{in}\;\mathrm{initial}\;\mathrm{sample}}$$

The protein content was quantified using the Bradford assay.

### 2.11. Sensory Analysis of the Yogurt Samples

The sensory analysis of the yogurt was confirmed with 21 trained evaluators using a random design. The sample without CSP was served as the control, and the yogurt samples containing CSP of different levels were served in transparent plastic plates, each labeled with a random number. Evaluators assessed five attributes, including taste, appearance, color, aroma, and overall acceptability. The presentation order of the samples was randomly coded according to the scoring criteria of Podder et al. [31].

### 2.12. Statistical Analysis

Statistical analysis was carried out using SPSS (version 26.0; SPSS Inc., Chicago, IL, USA). All experiments were conducted three or more times. Data are represented as the mean ± standard deviation (SD). The differences between groups were compared using two-way ANOVA, and means were further assessed with the least significant difference (LSD) test (*p* < 0.05).

## 3. Results and Discussion

### 3.1. Titratable Acidity (TA) and pH Analysis of the Yogurt Samples

#### 3.1.1. Changes in TA and pH of All Samples During Fermentation Processing

The addition of coriander seed powder (CSP) evidently affected the properties of the yogurt. As presented in Table 1, there was a notable difference (*p* < 0.05) for pH and TA during the fermentation process. The pH of CSP-fortified yogurts decreased significantly, while TA increased markedly from 3 h onward. Compared to the control, yogurts containing CSP exhibited significantly lower pH and higher TA levels (*p* < 0.05). Lactic acid bacteria (LAB), primarily *Lactobacillus delbrueckii* subsp. *bulgaricus* and *Streptococcus thermophilus*, drove yogurt fermentation. This enhancement in acidity is attributed to LAB metabolizing lactose into lactic acid, resulting in a decrease in pH and an incremental increase in TA [15]. Notably, the flavor compounds in coriander seeds may enhance LAB metabolic activity, accelerating lactic acid production, which in turn intensifies pH reduction and TA elevation [32,33]. These findings align with those demonstrated by Nakov et al. [34], demonstrating that the supplement of chia seeds brought about a diminution in the pH. Specifically, the pH of the control sample declined by 0.17 units, corresponding to a reduction of 4.05%. Yogurt formulations with 5% and 10% chia seed content exhibited decreases in pH of 0.08 and 0.13 units, equivalent to reductions of 1.90% and 3.07%, respectively. The increase in TA may be owed to the fermentation process, during which lactic acid and some organic acids (e.g., butyric acid, acetic acid, citric acid, and formic acid) are continuously produced, thereby elevating the TA value.

#### 3.1.2. TA and pH Changes in the Yogurt Samples During Storage Periods

Table 2 presents the pH and TA of yogurt from different groups during the storage period. Throughout the storage period, a significant drop in pH and an enhancement in TA were observed (*p* < 0.05). The reduction in pH and rise in TA became more pronounced with increasing concentrations of coriander seed powder (CSP). These findings are consistent with previous studies on yogurts supplemented with various dried plant materials, such as bilberry pomace powder, in which the pH of yogurt with added bilberry pomace powder was reduced by 0.3 units, compared to the control (4.70). This phenomenon is owed to the continuous accumulation of lactic acid derived from lactic acid bacteria fermentation [34,35]. During refrigerated storage, samples containing CSP exhibited lower pH values and higher TA levels compared to the control, reflecting a consistent trend between decreasing pH and increasing acidity.

### 3.2. Syneresis of Yogurt Samples

Syneresis is caused by the separation of whey from the yogurt gel matrix during storage periods. During the storage of yogurt, the degree of syneresis can reflect the quality and overall acceptability of yogurt [36]. In the current research, coriander seed powder (CSP) supplementation evidently affected the trend of syneresis in yogurt samples (Table 3). All groups demonstrated a significant decreasing trend in syneresis (*p* < 0.05). As suggested by Arab et al. [37], the casein-based network may undergo structural rearrangement, leading to contraction and subsequent whey expulsion. Notably, the yogurt samples fortified with 0.5% and 0.3% exhibited significantly lower syneresis, compared with the control group (*p* < 0.05). These improvements are attributed to the fact that the addition of CSP improves the water-holding capacity of whey protein, which further promotes the formation of a compact three-dimensional protein network with enhanced water-holding capacity [38]. A study provided by Guven et al. (2005) [39] demonstrated a 3.34% reduction in syneresis in yogurt samples supplemented with Moringa pod powder (MPP), compared with the control sample. These quality improvements can be due to the high dietary fiber content of MPP [39]. Furthermore, interactions between milk proteins and polyphenol-rich compounds facilitate the formation of stable complexes, strengthen water-holding capacity and casein network stability, and ultimately reduce syneresis in dairy products [40].

### 3.3. Texture and Rheological Properties of Yogurt Samples Containing CSP

The texture attribute is a dominant element impacting the quality of yogurt and overall acceptance of consumers. Table 4 shows the texture profile analysis (hardness, springiness, and adhesiveness) of yogurt samples supplemented with varying concentrations of coriander seed powder (CSP). During storage periods, CSP addition clearly influenced the texture of yogurt samples (*p* < 0.05). Hardness, a primary texture indicator, progressively increased during refrigerated storage, reflecting a structural reorganization of the protein matrix. As noted by Zang et al. [41], the enhanced casein–casein interaction promoted the formation of a denser gel network, so that the appearance of yogurt samples showed higher hardness. It is worth noting that the 0.5% CSP supplementation group exhibited significantly lower hardness; however, the yogurt samples with the 0.3% CSP still demonstrated higher hardness, compared with the control sample during the storage period (*p* < 0.05). Both springiness and adhesiveness displayed an evidently increasing trend and reached the peak on the 7th day of storage (*p* < 0.05) (Table 4). These reductions in springiness and adhesiveness of the yogurt samples in the late-stage period are attributed to progressive degradation of the protein network in the acidic environment. Long-term storage reduces the lactic acid bacteria activity and prevents further acidification, while the accumulation of lactic acid disrupts protein interactions [42]. The decrease in these indexes corresponds to the syneresis phenomenon of yogurt samples, and the whey precipitation also leads to the decline in springiness and adhesiveness. These findings are consistent with the observed correlation between texture properties and whey separation dynamics [43].

**Figure 1:**
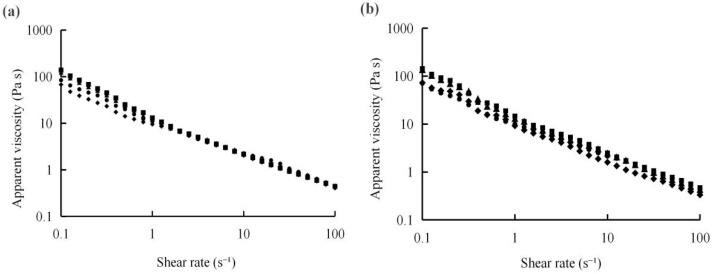

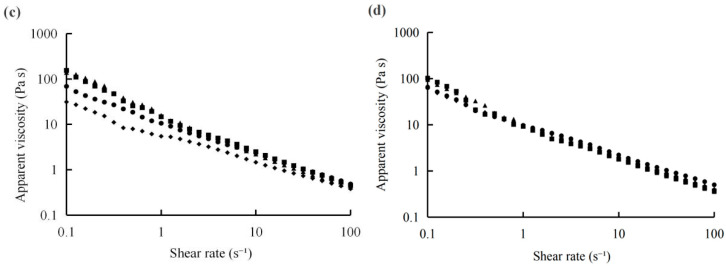
Apparent viscosity of the spoonable yoghurt assayed at 1 (**a**), 7 (**b**), 14 (**c**), and 21 days (**d**). Control (●), 0.1% CSP (▲), 0.3% CSP (■), and 0.5% CSP (◆). CSP—coriander seed powder.

**Figure 2:**
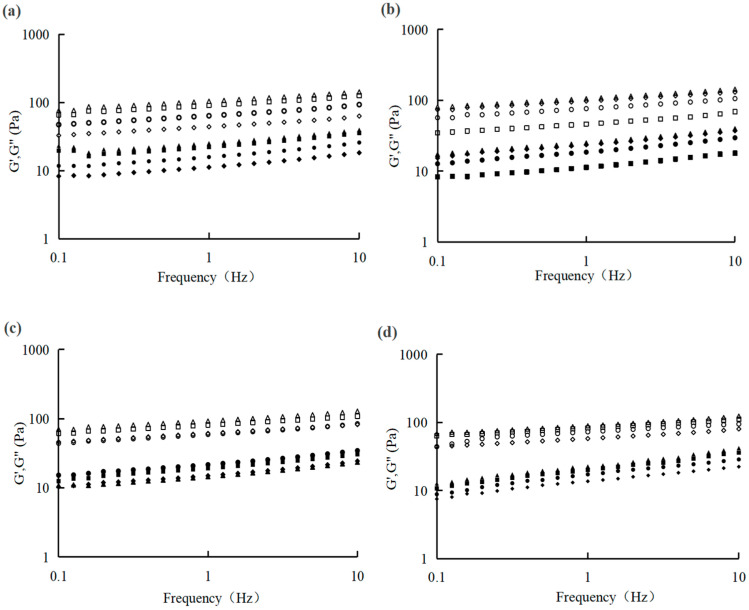
G′ and G″ of the spoonable yoghurt assayed at 1 (**a**), 7 (**b**), 14 (**c**), and 21 days (**d**). G′: Control (○), 0.1% CSP (△), 0.3% CSP (□), and 0.5% CSP (◇). G″: Control (●), 0.1% CSP (▲), 0.3% CSP (■), and 0.5% CSP (◆). CSP—coriander seed powder.

The evaluation of rheological parameters (such as G′, G″, and apparent viscosity) reflects the changes in the internal structure of the yogurt samples (Figure 1 and Figure 2). The apparent viscosity of these samples reduced with shear rate increasing, showing shear-thinning behaviour. Results exhibited that the samples with 1% CSP demonstrated higher viscosity values, compared with the other samples, the result is shown in Figure 1. G′ reflects the elastic energy stored in the sample; meanwhile, G″ presents the viscous energy dissipated during deformation, serving as a major determination indicator of viscosity behavior [44]. The rheological performance of the yogurt during the storage period is shown in Figure 2. The results demonstrated that the G′ and G″ of all samples with 0.3% CSP were higher on the 1st, 7th, and 21st day, compared with the control and 0.5% addition group, showing stable viscosity behavior. This phenomenon may be due to the polyphenol found in CSP through electrostatic, hydrogen bonding, and spatial interactions, thus forming a casein–polyphenol complex and strengthening the yogurt gel network stability [44,45]. During the whole storage period, the values of G′ and G″ fluctuated, which influences the rheological properties of the yogurt samples. However, all yogurt samples with CSP retained the characteristics of a weak viscoelastic gel (G′ > G″), exhibiting a predominant elastic structure (solid-like behavior). This structure effectively kept water in the system, thereby enhancing storage stability. These discoveries are in accordance with the results demonstrated by Wang et al. [46].

### 3.4. Antioxidant Activity of the Yogurt

**Figure 3:**
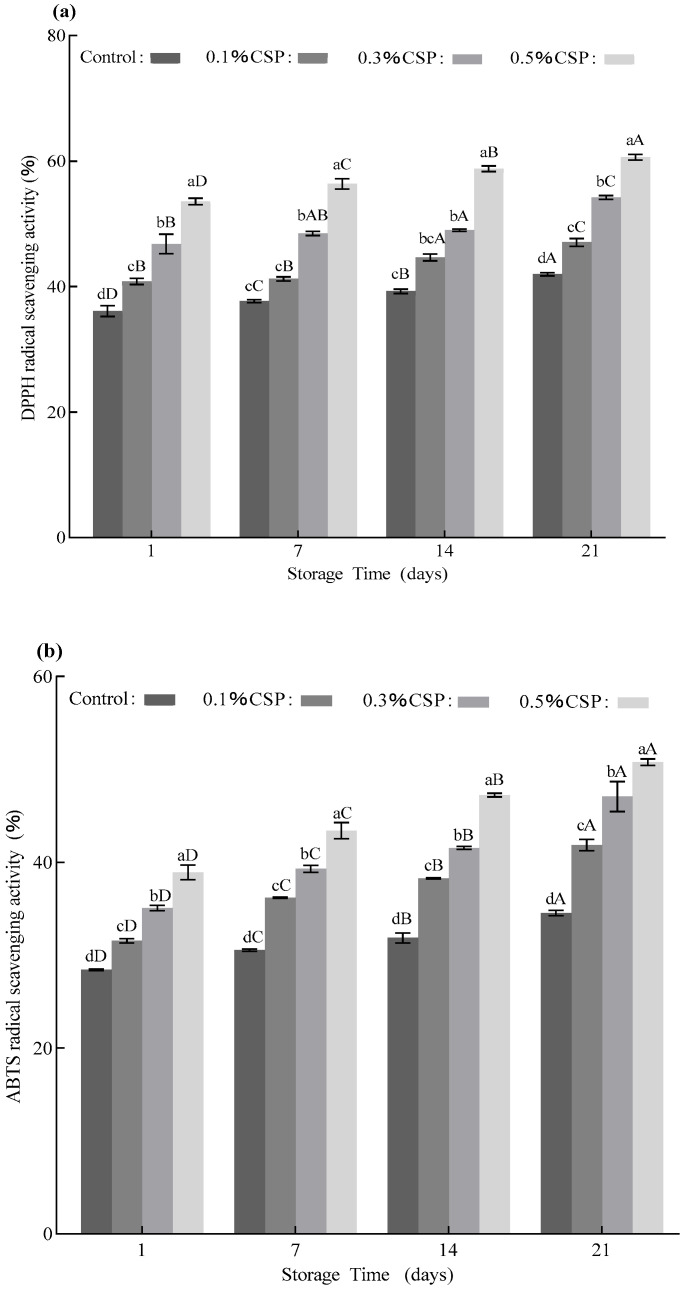
The antioxidant activity changes in the yogurt samples. (**a**) Changes in DPPH levels of the yogurt samples during storage; (**b**) changes in ABTS content of the yogurt samples during storage period. CSP—coriander seed powder. Note: Two-way ANOVA was performed. Different letters in the figure indicate significant differences between groups in the analysis results (*p* < 0.05).

The DPPH-radical scavenging activity demonstrated a significant concentration- and time-dependent enhancement (Figure 3a). Higher concentrations of coriander seed powder were associated with progressively greater scavenging activity. On the 1st day, the 0.3%-fortified CSP group exhibited higher activity (39.25%), compared to the control group (36.11%). The scavenging activity continued to increase across all groups, reaching its maximum improvement (0.3% CSP group, 19.54%) on the 21st day, compared to the control group (17.46%). This enhancement during storage suggests the sustained release of bioactive small molecules or the formation of an effective combination with casein, which is in agreement with results studied by Sheikh et al. [47]. Therein, the antioxidant character of the control sample ranged from 26.43% to 33.37% during the storage period of 28 days, whereas the yogurt samples with added 6% white mulberry fruit powder (WMFP) showed enhanced activity levels (from 51.50% to 57.59%).

A concentration-dependent effect on ABTS^+^-radical scavenging activity was also observed, as illustrated in Figure 3b. On the first day, the ABTS^+^ clearance rate rose from 28.42% to 35.07% as the concentration of coriander seed powder rose from 0.1% to 0.3%. During storage, the ABTS^+^ scavenging ability continued to improve, and the 0.3%-fortified CSP group demonstrated the most notable enhancement, which increased by 11.99% on the 21st, compared to the first day. The 0.3% and 0.5% addition groups maintained higher and more stable antioxidant activity during the storage period, compared with the control and the 0.1% addition group. These findings suggest that CSP addition not only strengthens the initial antioxidant feature of the yogurt samples but also contributes to their antioxidant stability during long-term storage.

### 3.5. Contents of Total Polyphenols and Flavonoids in the Yogurt

The addition of coriander seed powder significantly enhanced the total phenolic content (TPC) of the yogurt (*p* < 0.05) (Table 5). On the 21st day, the yogurt samples fortified with 0.3% CSP exhibited an increase of 1.85 mg GAE/100 g in total phenolic content, compared with the control. During the 21-day storage period, the TPC of the yogurt samples with CSP significantly remained higher than that of the control group (*p* < 0.05). This dose-dependent increase is consistent with expectations, as coriander seeds are abundant in phenolic substances, containing flavonoids and phenolic acids [48]. This enhancement of TPC in the yogurt samples is consistent with research findings on yogurt products fortified with other polyphenol-rich plant extracts. In a previous study, the yogurt sample without lotus seed powder exhibited a lower TPC, approximately 41.33 mg GAE/g, whereas the sample supplemented with lotus seed powder showed a notably higher TPC value of 80.33 mg GAE/g [49]. This relative stability suggests that polyphenol-rich substances from natural products with non-covalent interactions between proteins, like hydrophobic bonds, van der Waals interactions, electrostatic interactions, and hydrogen bonding, which can enhance product oxidative stability and reduce polyphenol degradation [50,51]. The evident increase in TPC represents a key functional benefit of fortification with coriander seed powder. Phenolic compounds are potent antioxidants associated with various health benefits, including reduced oxidative stress and potential mitigation of chronic disease risks. The addition of bioactive ingredients in yogurt provides a feasible strategy for developing functional dairy products with enhanced biological activity characteristics. Notably, compared to the total flavonoid content (TFC) trend, TPC further increased during the 21-day storage period. In contrast, TFC levels increased with the concentration of coriander seed powder but gradually lowered during storage. After 21 days of storage at 4 °C, the TFC of all yogurt groups decreased, including the control group. These findings are consistent with reports of flavonoid degradation in plant-based yogurt products during refrigeration [52].

### 3.6. In Vitro Protein Digestion of the Yogurt Samples

**Figure 4:**
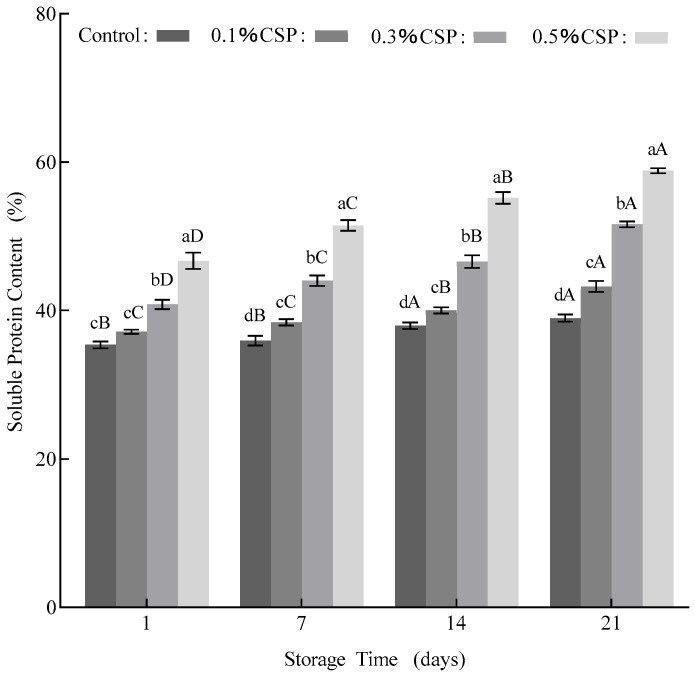
In vitro protein digestion results of the yogurt samples. CSP—coriander seed powder. Note: Two-way ANOVA was performed. Different letters in the figure indicate significant differences between groups in the analysis results (*p* < 0.05).

The evaluation of protein digestibility can effectively reflect the in vivo utilization rate of absorbed protein [10]. As shown in Figure 4, the protein digestibility of the yogurt fortified with coriander seed powder significantly increased with prolonged digestion time and consistently remained higher than that of the control yogurt sample (*p* < 0.05). This enhanced digestibility can be attributed to the smaller lipid droplet size in the fortified yogurt, which expands the surface area for protease interaction and activity [53]. Additionally, during digestion, the dietary fiber in coriander seed powder absorbs water, swells, and contributes to satiety through gastric distension. Simultaneously, the bioactive compounds in the coriander seed powder may be gradually released during fermentation processing or metabolized into smaller, more readily absorbable molecules by digestive enzymes, thereby enhancing the digestion rate [54]. Collectively, these results demonstrate that the incorporation of coriander seed powder into the yogurt samples improves its protein digestibility during gastrointestinal transit.

### 3.7. Sensory Assessment of the Yogurt Samples

**Figure 5:**
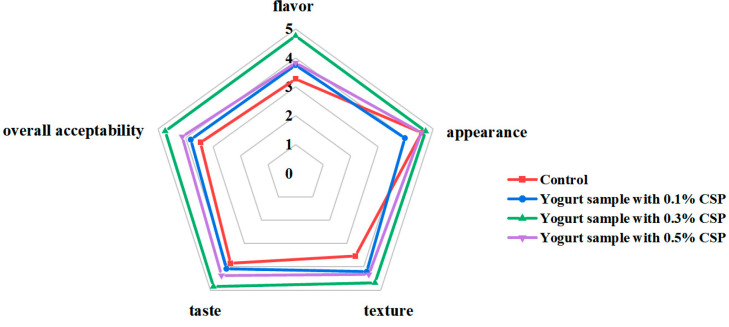
Graphical representation of sensory evaluation of all samples.

**Figure 6:**
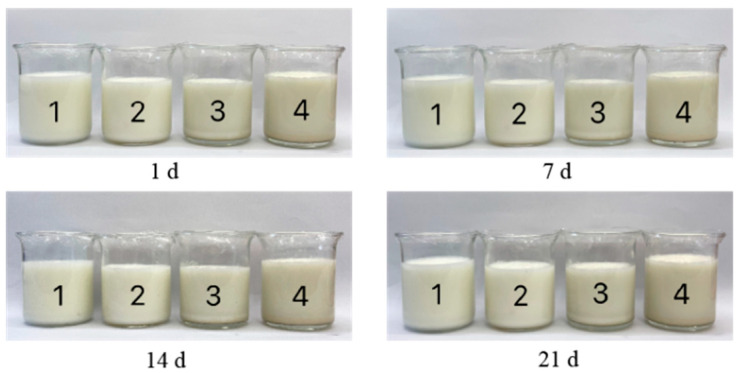
Pictures of refrigerated yogurt samples with different added amounts of CSP for 1, 7, 14, and 21 days. The numbers 1, 2, 3, and 4 represent the control sample, the yogurt sample with 0.1% CSP, the yogurt sample containing 0.3% CSP, and the yogurt sample including 0.5% CSP, respectively. CSP—coriander seed powder.

As shown in Figure 5, the yogurt samples containing 0.3% CSP achieved the highest preference for all sensory indexes, including flavor, appearance, texture, and overall acceptability. However, the yogurt samples with 0.1% and 0.5% CSP displayed the lowest acceptability. The control samples appeared visually white, with a characteristic lactic acid fermentation flavor and a smooth, delicate taste. However, as the CSP concentration increased to 0.5%, the color of the yogurt samples turned deeper gray, accompanied by subtle fermentation odor and a more granular texture (Figure 6). Sensory evaluation scores for the yogurt samples containing 0.1% CSP were obviously lower than those for the 0.3% CSP group and the control (*p* < 0.05). The breakdown of milk fat is a crucial step in the development of yogurt flavor [55]. Fat degradation produces fatty acids, including short-chain aliphatic acids (e.g., hexanoic acid and butyric acid), which impart distinct flavors. These fatty acids not only contribute directly to flavor but can also undergo esterification to form ester compounds, enriching the yogurt’s aroma. The addition of CSP did not result in any undesirable flavor. However, some evaluators noted that higher concentrations of CSP (especially 0.5%) caused a noticeable decrease in color appeal, an increase in graininess, and a less refined taste compared to the other samples.

## 4. Conclusions

The incorporation of coriander seed powder (CSP) significantly accelerated the increase in acidity and notably shortened the fermentation time. It was observed that 0.3% CSP addition levels substantially enhanced the texture and rheological properties and decreased the syneresis of the solidified yogurt. Moreover, higher concentrations of CSP progressively improved the antioxidant feature of the yogurt. After 21 days of cold storage, the DPPH clearance rate reached 60.63%, while the ABTS^+^-radical scavenging rate was 50.77%. The total phenolic concentration (5.33 ± 0.15 mg GAE/100 g) and total flavonoid level (5.28 ± 0.01 mg CE/100 g) of the yogurt sample with 0.5% CSP peaked in the final stage of the storage period. The obtained results confirm that CSP, a rich source of polyphenols, coumarin, and flavonoids, provides a strong prebiotic effect and significantly enhances antioxidant activity in yogurt. Coriander seeds are abundant in dietary fiber, which can be used as a prime source of prebiotics. Prebiotics can effectively promote the growth and activity of beneficial microorganisms. Meanwhile, these microorganisms effectively produce short-chain fatty acids, including acetic acid, propionic acid, and butyric acid, by metabolizing prebiotics, thus providing nutrition for the intestinal environment and maintaining the balance of the microorganisms. The addition of CSP obviously improved the casein gel network structure, resulting in a more continuous, smooth, and uniform microstructure during the cold storage period. This phenomenon can be attributed to the natural polysaccharose and organic acid in CSP, promoting a notable proliferation of lactic acid bacteria. In vitro digestion experiments demonstrated that the yogurt supplemented with CSP exhibited improved protein digestibility. Sensory evaluation results indicated that yogurt containing 0.3% CSP not only improved the overall quality but was also the most favored by evaluators. Coriander seed is rich in dietary fiber, fatty acids, polysaccharides, vitamins, and high-quality bioactive compounds, including polyphenols, flavonoids, and phenolic acids. Therefore, its application in fermented dairy products possesses the potential to reinforce immune function and facilitate overall health. In conclusion, based on its perfect performance in improving the physicochemical properties, optimizing microstructure, and enhancing sensory quality, the addition level of 0.3% CSP in yogurt is recommended for the development of functional fermentation products. While this study primarily assessed the effects of CSP on physicochemical properties and sensory quality of the yogurt, the specific mechanisms of the impact of the fermentation process on polyphenol levels have not been well evaluated. Furthermore, the particle size and solubility of coriander seed powder may affect the quality of yogurt. During the processing of yogurt, coriander seed powder at a high level could not be completely dissolved or dispersed, which may lead to uneven texture and influence the overall quality. In the future, the effects of coriander seed powder on the growth of lactic acid bacteria, acid generation rate, or strain activity can be further assessed. In order to improve the dispersibility and stability of coriander seed powder in dairy products, ultrafine grinding or other processing techniques should be further explored. This is also the main direction of our future research.

## Figures and Tables

**Table 1:** Changes in the pH value and TA of all samples during the fermentation process.

Parameters	Fermentation Time (h)	Control	0.1% CSP	0.3% CSP	0.5% CSP
pH	0	6.95 ± 0.07 ^aA^	6.80 ± 0.03 ^abA^	6.67 ± 0.03 ^abA^	6.62 ± 0.04 ^bB^
	1	6.73 ± 0.03 ^aA^	6.61 ± 0.02 ^abA^	6.51 ± 0.14 ^bA^	6.61 ± 0.02 ^abA^
	2	6.69 ± 0.02 ^aA^	6.59 ± 0.01 ^bA^	6.56 ± 0.02 ^bA^	6.55 ± 0.00 ^bAB^
	3	6.14 ± 0.01 ^aB^	5.81 ± 0.03 ^bB^	5.53 ± 0.02 ^cB^	5.53 ± 0.01 ^cC^
	4	5.80 ± 0.02 ^aC^	5.20 ± 0.02 ^bC^	5.10 ± 0.09 ^bcC^	5.03 ± 0.02 ^cD^
	5	4.71 ± 0.01 ^aD^	4.71 ± 0.03 ^aD^	4.51 ± 0.00 ^bD^	4.52 ± 0.01 ^bE^
Titratable acidity (°T)	0	6.73 ± 0.32 ^bE^	7.33 ± 0.45 ^bD^	8.43 ± 0.41 ^aD^	9.03 ± 0.05 ^aC^
	1	7.00 ± 0.26 ^bDE^	7.46 ± 0.64 ^bD^	8.16 ± 0.15 ^abD^	9.76 ± 1.07 ^aC^
	2	7.63 ± 0.05 ^bD^	7.86 ± 0.41 ^abD^	8.50 ± 0.50 ^abD^	10.26 ± 1.77 ^aC^
	3	11.53 ± 0.35 ^dC^	16.26 ± 0.35 ^cC^	20.63 ± 0.15 ^bC^	21.66 ± 0.51 ^aB^
	4	14.83 ± 0.05 ^dB^	23.43 ± 0.25 ^cB^	30.03 ± 0.95 ^bB^	34.40 ± 2.10 ^aA^
	5	16.56 ± 0.25 ^cA^	26.86 ± 0.40 ^bA^	33.93 ± 0.45 ^aA^	32.20 ± 1.56 ^aA^

Values are exhibited as mean ± SD (*n* = 3). ^a–d^ Means displayed in the same row by different lowercase letters for the same parameter are clearly different (*p* < 0.05). ^A–E^ Means showed by different uppercase letters in the same column for the same parameter are evidently different (*p* < 0.05).CSP—coriander seed powder.

**Table 2:** Changes in pH and TA of all samples during storage period.

Parameters	Storage Time (Days)	Control	0.1% CSP	0.3% CSP	0.5% CSP
pH	1	4.71 ± 0.00 ^aB^	4.60 ± 0.10 ^abAB^	4.41 ± 0.00 ^bcB^	4.34 ± 0.02 ^cB^
	7	4.64 ± 0.01 ^aA^	4.54 ± 0.02 ^aA^	4.45 ± 0.02 ^bAB^	4.30 ± 0.06 ^cB^
	14	4.59 ± 0.01 ^aA^	4.52 ± 0.02 ^bA^	4.38 ± 0.03 ^cA^	4.21 ± 0.01 ^dA^
	21	4.40 ± 0.04 ^aC^	4.31 ± 0.05 ^abB^	4.21 ± 0.04 ^bC^	4.06 ± 0.03 ^cC^
Titratable acidity (°T)	1	34.86 ± 1.13 ^cD^	36.63 ± 0.83 ^cD^	45.06 ± 1.09 ^aBC^	47.20 ± 0.65 ^bA^
	7	38.80 ± 0.60 ^bC^	40.86 ± 1.06 ^bC^	43.70 ± 1.22 ^aC^	49.46 ± 1.22 ^bAB^
	14	44.93 ± 0.77 ^aB^	45.26 ± 0.50 ^aB^	46.80 ± 1.17 ^aB^	51.00 ± 0.81 ^bB^
	21	51.63 ± 0.70 ^cA^	54.06 ± 0.45 ^bA^	57.20 ± 0.88 ^aA^	55.90 ± 0.45 ^dC^

Values are expressed as mean SD (*n* = 3). ^a–d^ Means followed by different lowercase letters in the same row are obviously different for the same parameter (*p* < 0.05). ^A–D^ Means followed by different uppercase letters in the same column are markedly different for the same parameter (*p* < 0.05). CSP—coriander seed powder.

**Table 3:** Syneresis changes in yogurt samples during storage.

Parameters	Storage Time (Days)	Control	0.1% CSP	0.3% CSP	0.5% CSP
Syneresis (%)	1	33.71 ± 2.31 ^aC^	27.68 ± 3.67 ^abA^	23.11 ± 0.83 ^bC^	21.92 ± 1.78 ^bA^
	7	40.24 ± 1.91 ^aB^	31.14 ± 2.97 ^bA^	25.08 ± 0.40 ^cBC^	23.07 ± 0.98 ^cA^
	14	44.09 ± 1.17 ^aB^	32.92 ± 0.68 ^bA^	27.01 ± 1.90 ^cAB^	23.93 ± 0.15 ^dA^
	21	49.64 ± 2.11 ^aA^	33.00 ± 1.05 ^bA^	28.50 ± 0.19 ^cA^	24.05 ± 1.51 ^dA^

Values are expressed as mean ± SD (*n* = 3). ^a–d^ Means followed in the same row by diverse lowercase letters are markedly different (*p* < 0.05). ^A–C^ Means followed by distinct uppercase letters in the same column are significantly different for the same parameter (*p* < 0.05). CSP—coriander seed powder.

**Table 4:** Texture indices of yogurt during cold storage.

Parameters	Samples				
	Storage Time (Days)	Control	0.1% CSP	0.3% CSP	0.5% CSP
Hardness (N)	1	0.87 ± 0.06 ^bC^	1.32 ± 0.18 ^aA^	1.28 ± 0.16 ^aA^	0.64 ± 0.16 ^bB^
	7	0.90 ± 0.09 ^bC^	1.32 ± 0.01 ^aA^	1.32 ± 0.06 ^aA^	0.92 ± 0.02 ^bA^
	14	1.18 ± 0.01 ^bB^	1.44 ± 0.08 ^aA^	1.41 ± 0.05 ^aA^	0.92 ± 0.01 ^cA^
	21	1.43 ± 0.01 ^aA^	1.51 ± 0.25 ^aA^	1.48 ± 0.09 ^aA^	0.93 ± 0.02 ^bA^
Springiness (mm)	1	5.71 ± 0.03 ^aC^	5.57 ± 1.54 ^aA^	6.18 ± 0.13 ^aA^	6.26 ± 0.03 ^aA^
	7	5.83 ± 0.13 ^bBC^	5.68 ± 0.01 ^bA^	6.02 ± 1.08 ^abA^	7.79 ± 0.90 ^aA^
	14	7.44 ± 0.98 ^aA^	5.35 ± 0.01 ^aA^	5.70 ± 0.77 ^aA^	7.48 ± 1.37 ^aA^
	21	7.09 ± 0.08 ^aAB^	5.25 ± 0.22 ^bA^	5.60 ± 0.03 ^abA^	7.23 ± 1.28 ^aA^
Adhesiveness (N.mm)	1	1.22 ± 0.03 ^aC^	1.40 ± 0.37 ^aB^	1.46 ± 0.28 ^aA^	0.79 ± 0.51 ^aB^
	7	1.29 ± 0.09 ^bBC^	1.45 ± 0.04 ^bB^	1.87 ± 0.03 ^aA^	1.47 ± 0.17 ^cAB^
	14	1.61 ± 0.15 ^aA^	1.79 ± 0.01 ^aA^	1.88 ± 0.21 ^aA^	1.58 ± 0.05 ^aA^
	21	1.39 ± 0.08 ^bB^	1.42 ± 0.02 ^bB^	1.95 ± 0.13 ^aA^	1.40 ± 0.14 ^bAB^

Values are expressed as mean ± SD (*n* = 3). ^a–c^ Means followed by different lowercase letters in the same row are markedly different (*p* < 0.05). ^A–C^ Means followed by different uppercase letters in the same column for the same arguments are clearly different (*p* < 0.05). CSP—coriander seed powder.

**Table 5:** Changes in TPC and TFC of yogurt during storage.

Parameters	Storage Time (Days)	Control	0.1% CSP	0.3% CSP	0.5% CSP
Total phenol content (mg/100 g)	1	1.65 ± 0.14 ^aD^	1.87 ± 0.24 ^aD^	1.93 ± 0.08 ^aC^	2.00 ± 0.05 ^aB^
	7	2.27 ± 0.03 ^bC^	2.52 ± 0.15 ^bC^	3.16 ± 0.10 ^abB^	4.79 ± 1.59 ^aA^
	14	2.91 ± 0.02 ^bB^	3.81 ± 0.03 ^bB^	5.04 ± 0.91 ^aA^	5.54 ± 0.04 ^aA^
	21	3.48 ± 0.02 ^cA^	5.05 ± 0.18 ^bA^	5.33 ± 0.15 ^bA^	6.29 ± 0.06 ^aA^
Total flavonoid content (mg/100 g)	1	4.33 ± 0.21 ^bA^	4.43 ± 0.24 ^bA^	5.28 ± 0.01 ^aA^	5.53 ± 0.15 ^aA^
	7	3.65 ± 0.31 ^bB^	4.11 ± 0.07 ^abA^	4.30 ± 0.22 ^abB^	4.96 ± 0.53 ^aAB^
	14	2.82 ± 0.03 ^dC^	3.52 ± 0.10 ^cB^	3.79 ± 0.05 ^bC^	4.28 ± 0.03 ^aBC^
	21	2.36 ± 0.03 ^dC^	3.11 ± 0.13 ^cC^	3.58 ± 0.08 ^bC^	3.98 ± 0.03 ^aC^

Values are expressed as mean SD (*n* = 3). ^a–d^ Means followed in the same row by different lowercase letters for the same parameter are obviously different (*p* < 0.05). ^A–D^ Means followed by different uppercase letters in the same column for the same parameter are markedly different (*p* < 0.05). CSP—coriander seed powder.

## Data Availability

The original contributions presented in this study are included in the article. Further inquiries can be directed to the corresponding authors.
